# Outcomes and Predictors of Mortality in Patients With KPC-Kp Infections Treated With Meropenem Vaborbactam: An Observational Multicenter Study

**DOI:** 10.1093/ofid/ofae273

**Published:** 2024-05-08

**Authors:** Mario Tumbarello, Francesca Raffaelli, Maddalena Giannella, Gennaro De Pascale, Antonio Cascio, Francesco Giuseppe De Rosa, Anna Maria Cattelan, Alessandra Oliva, Annalisa Saracino, Matteo Bassetti, Cristina Mussini, Roberto Luzzati, Alessandro Capone, Liana Signorini, Michele Bartoletti, Margherita Sambo, Loredana Sarmati, Spinello Antinori, Alessandra Mularoni, Carlo Tascini, Alberto Corona, Renato Pascale, Raffaella Rubino, Silvia Corcione, Maria Mazzitelli, Gabriele Giuliano, Antonio Lovecchio, Davide Fiore Bavaro, Marianna Meschiari, Francesca Montagnani, Massimiliano Fabbiani, Ilaria De Benedetto, Massimo Antonelli, Mario Venditti, Pierluigi Viale

**Affiliations:** Department of Medical Biotechnologies, University of Siena, Siena, Italy; Infectious and Tropical Diseases Unit, Azienda Ospedaliero Universitaria Senese, Siena, Italy; Dipartimento di Scienze di Laboratorio e Infettivologiche, Fondazione Policlinico Universitario A. Gemelli IRCCS, Roma, Italy; Department of Medical and Surgical Sciences, University of Bologna, Bologna, Italy; Dipartimento di Scienze Biotecnologiche di base, Cliniche Intensivologiche e Perioperatorie, Università Cattolica del Sacro Cuore, Rome, Italy; Dipartimento di Scienza dell'Emergenza, Anestesiologiche e della Rianimazione, Fondazione Policlinico Universitario A. Gemelli IRCCS, Roma, Italy; Department of Health Promotion, Mother and Child Care, Internal Medicine and Medical Specialties “G D'Alessandro”, University of Palermo, Palermo, Italy; Infectious and Tropical Disease Unit, AOU Policlinico “P. Giaccone” Palermo, Italy; Infectious diseases, Department of Medical Sciences, University of Turin, Torino, Italy; Infectious and Tropical Diseases Unit, Padua University Hospital, Padova, Italy; Department of Molecular Medicine, University of Padova, Padova, Italy; Dipartimento di Sanità Pubblica e Malattie Infettive, Università Sapienza, Roma, Italy; Operative Unit of Infectious Diseases, Hospital-University Polyclinic of Bari, Bari, Italy; Infectious Diseases Unit, Ospedale Policlinico San Martino - IRCCS, Genoa, Italy; Department of Health Sciences (DISSAL), University of Genoa, Genoa, Italy; Clinica delle Malattie Infettive, Università di Modena e Reggio Emilia, Modena, Italy; Infectious Diseases Unit, University Hospital of Trieste, Trieste, Italy; Infezioni Sistemiche ed Immunodepresso, National Institute for Infectious Disease L. Spallanzani, Roma, Italy; UOC Malattie Infettive, Spedali Civili di Brescia, Brescia, Italy; Department of Biomedical Sciences, Humanitas University, Pieve Emanuele, Milan, Italy; Infectious Diseases Unit, IRCCS Humanitas Research Hospital, Milan, Italy; Department of Medical Biotechnologies, University of Siena, Siena, Italy; Infectious and Tropical Diseases Unit, Azienda Ospedaliero Universitaria Senese, Siena, Italy; Clinical Infectious Diseases, Department of System Medicine, Tor Vergata University, Roma Italy; Dipartiment of Scienze Biomediche e Cliniche L. Sacco, Università degli Studi di Milano Polo Universitario, Milano, Italy; Infectious Diseases Unit, ISMETT-IRCCS Istituto Mediterraneo per i Trapianti e Terapie ad Alta Specializzazione, Palermo, Italy; Infectious Disease Clinic, DAME (Department of Medicine) University of Udine, Udine Italy; ICU, Surgical Theatre & Emergency Department, ASST Valcamonica, Breno Italy; Department of Medical and Surgical Sciences, University of Bologna, Bologna, Italy; Infectious and Tropical Disease Unit, AOU Policlinico “P. Giaccone” Palermo, Italy; Infectious diseases, Department of Medical Sciences, University of Turin, Torino, Italy; Infectious and Tropical Diseases Unit, Padua University Hospital, Padova, Italy; Infectious and Tropical Diseases Unit, Azienda Ospedaliero Universitaria Senese, Siena, Italy; Infectious Diseases Unit, University Hospital of Trieste, Trieste, Italy; Operative Unit of Infectious Diseases, Hospital-University Polyclinic of Bari, Bari, Italy; Clinica delle Malattie Infettive, Università di Modena e Reggio Emilia, Modena, Italy; Department of Medical Biotechnologies, University of Siena, Siena, Italy; Infectious and Tropical Diseases Unit, Azienda Ospedaliero Universitaria Senese, Siena, Italy; Department of Medical Biotechnologies, University of Siena, Siena, Italy; Infectious and Tropical Diseases Unit, Azienda Ospedaliero Universitaria Senese, Siena, Italy; Infectious diseases, Department of Medical Sciences, University of Turin, Torino, Italy; Dipartimento di Scienze Biotecnologiche di base, Cliniche Intensivologiche e Perioperatorie, Università Cattolica del Sacro Cuore, Rome, Italy; Dipartimento di Scienza dell'Emergenza, Anestesiologiche e della Rianimazione, Fondazione Policlinico Universitario A. Gemelli IRCCS, Roma, Italy; Dipartimento di Sanità Pubblica e Malattie Infettive, Università Sapienza, Roma, Italy; Department of Medical and Surgical Sciences, University of Bologna, Bologna, Italy

**Keywords:** carbapenemases, KPC-producing Klebsiella pneumoniae, meropenem-vaborbactam, ceftazidime-avibactam resistance, bloodstream infection

## Abstract

**Background:**

Meropenem-vaborbactam is a recent and promising option for the treatment of KPC-producing *Klebsiella pneumoniae* (KPC-Kp) infections, including those resistant to ceftazidime-avibactam.

**Methods:**

We conducted a retrospective analysis of observational data from 19 Italian hospitals on use and outcomes of patients treated with meropenem-vaborbactam for at least ≥24 hours for KPC-Kp infections. Crude and propensity-weighted multiple Cox regression models were performed to ascertain risk factors independently associated with 30-day mortality.

**Results:**

The cohort included 342 adults with bloodstream infections (n = 172) and nonbacteremic infections (n = 170), of which 107 were lower respiratory tract infections, 30 were complicated urinary tract infections, and 33 were infections involving other sites. Most infections (62.3%) were managed with meropenem-vaborbactam monotherapy, or in combination with at least 1 other active drug (usually fosfomycin, tigecycline, or gentamicin) (37.7%). The 30-day mortality rate was 31.6% (108/342). In multiple Cox regression model, 30-day mortality was independently associated with septic shock at infection onset, Charlson comorbidity index ≥ 3, dialysis, concomitant COVID-19, and INCREMENT score ≥ 8. Administration of meropenem-vaborbactam within 48 hours from infection onset was a negative predictor of mortality. All predictors, except administration of meropenem-vaborbactam within 48 hours, remained significant when the multiple Cox regression model was repeated after adjustment for the propensity score for receipt of combination therapy.

**Conclusions:**

Despite the limits of a retrospective study, the data derived from this multicenter cohort provide additional evidence on the efficacy of meropenem-vaborbactam in treating severe KPC-Kp infections, even when used as monotherapy.

Carbapenemase-producing *Klebsiella pneumoniae* (KPC-Kp) is mainly involved in the majority of carbapenem-resistant Enterobacterales (CRE) infections in Italy, configuring itself as a serious threat to public health and causing challenging-to-treat infections associated with high mortality rates in hospitalized patients [1–3]. Of concern is the increase in reports of ceftazidime/avibactam-resistant strains, considered the first-line option for the treatment of these infections [4–6].

Meropenem-vaborbactam, a broad-spectrum antimicrobial combined with a novel cyclic boronic acid β-lactamase inhibitor, with activity against bacteria producing KPC and other serine β-lactamases, was analyzed in the TANGO II clinical trial, which provided initial information on the treatment of CRE [7, 8]. More evidence has emerged from real-world observational studies that provide information on the use of meropenem-vaborbactam in infections caused by CRE, supporting the clinical efficacy of meropenem-vaborbactam, and showing no difference in clinical outcomes when analyzed in comparison to ceftazidime-avibactam, with a mortality rate between 7.5% and 24.3% and infection recurrence between 11% and 13% [9–15]. Moreover, a small study showed that clinical success rate of meropenem-vaborbactam is similar for infections caused by ceftazidime/avibactam-resistant isolates [12].

To increase the evidence from real-life experience and devise strategies for the optimal use of this new drug in the treatment of KPC-Kp infections, we retrospectively analyzed a wide body of observational national data on the postmarketing use and outcomes of meropenem-vaborbactam therapy for infections caused by KPC-Kp isolates.

## METHODS

### Study Design

The study involved a retrospective analysis of observational data on inpatients in 19 Italian hospitals (both academic and nonacademic) who received meropenem-vaborbactam for KPC-Kp infections between 1 January 2022 and 30 June 2023. Patients eligible for study cohort enrollment met the following criteria: (1) age ≥18 years at hospital admission; (2) culture-documented monomicrobial KPC-Kp infection; and (3) ≥ 24 hours of treatment with meropenem-vaborbactam, alone or with other antimicrobials with in vitro activity against the KPC-Kp isolate. Candidates were excluded if they had *K pneumoniae* isolates producing KPCs plus other carbapenemases, and in any case the initial isolates were resistant to meropenem-vaborbactam. Coordinators at each participating center reviewed enrolled patients’ electronic medical records and extracted data on the patients’ demographic and comorbidity profiles; epidemiological, clinical, and microbiological features of the infections; characteristics of the antimicrobial treatment regimens; and outcomes.

### Patient and Infection Profiles

The impact of comorbidities present at infection onset (the collection date of the index culture [ie, first culture yielding the study isolate]) was assessed in terms of individual conditions and Charlson Comorbidity Index [16]. Illness severity at infection onset was categorized based on the estimated mortality risk as indicated by the INCREMENT CPE score (low [<8 points]) versus high [≥8 points]) and the presence or absence of septic shock (ie, a subset of sepsis in which underlying circulatory and cellular/metabolic abnormalities are profound enough to substantially increase mortality) [17–20]. Infections were considered hospital-acquired if the index culture was collected more than 48 hours after hospital admission. Infections were classified as bloodstream infections (BSIs) if blood cultures were positive for a KPC-Kp strain. Nonbacteremic KPC-Kp infections were defined by (1) documented isolate of a KPC-Kp from cultures of nonblood samples (eg, urine, intra-abdominal wounds, sputum, bronchoalveolar lavage fluid); (2) no KPC-Kp-positive blood cultures during the index hospitalization; and (3) the presence of clinical and/or radiological signs of infection. Cases that failed to meet these criteria were classified as colonization and excluded from the analysis.

### Meropenem-vaborbactam Treatment and Follow-up

Meropenem-vaborbactam was administered intravenously at a standard dose (4 g [meropenem 2 g + vaborbactam 2 g] every 8 hours), with infusions delivered over a 3-hour period, and dosage was adjusted for renal impairment, following the manufacturer's recommendations.

Meropenem-vaborbactam treatment regimens classified as combination therapy included at least 1 other antimicrobial (administered for ≥72 hours) with in vitro activity against the patient's KPC-Kp isolate.

The primary endpoint of the study was all-cause mortality 30 days after infection onset. Secondary endpoints were infection relapse, development of in vitro meropenem-vaborbactam resistance and adverse events.

Infection relapse was defined as the onset of a second microbiologically documented KPC-Kp infection in a patient whose original infection had been classified as a clinical cure. Clinical cure was defined as clinical response to treatment with resolution of symptoms/signs of the infection on discontinuation of meropenem-vaborbactam therapy.

### Microbiology

Isolates were identified at the species level using matrix-assisted laser desorption ionization-time-of-flight mass spectrometer technology (MALDI Biotyper, Bruker Daltonics GmbH, Leipzig, Germany, or Vitek-MS, bioMérieux, Marcy l’Etoile, France) or, alternatively, biochemical identification methods (eg, Vitek 2, bioMérieux).

Each hospital conducted susceptibility testing according to its own protocols, mainly using Vitek 2 system (bioMérieux), Phoenix system (Becton, Dickinson and Company, Franklin Lakes, NJ) or the reference broth microdilution method. In some cases, gradient diffusion tests was adopted for meropenem-vaborbactam minimum inhibitory concentration determination. Susceptibility results were interpreted according to the most recent version of EUCAST clinical breakpoints [21].

Carbapenemases detection was performed from grown colonies using lateral flow immunoassay approach (eg, NG-Test CARBA 5 [NG Biotech, Guipry, France]; RESIST-3 O.O.K. K-SeT [Coris BioConcept, Gembloux, Belgium]) or by molecular methods (eazyplex SuperBug CRE assay [Amplex Diagnostics GmbH, Germany]; or the Xpert Carba-R assay [Cepheid, Sunnyvale, CA]).

### Statistical Analysis

Results are presented as means ± standard deviations or medians and interquartile ranges for continuous variables or as percentages for categorical variables. The Student *t* test and Mann-Whitney *U* test were used to compare normally and nonnormally distributed continuous variables, respectively. Categorical variables were evaluated with the chi-square or 2-tailed Fisher exact test. Odds ratios and 95% confidence intervals were calculated for all associations that emerged. Two-tailed tests were used to determine statistical significance reflected by a *P* value of <.05.

Multiple Cox regression models were performed to ascertain risk factors independently associated with 30-day mortality. A propensity score reflecting the likelihood of receiving combination rather than monotherapy was included in the model to balance baseline covariates predictive of treatment and control for confounding. The score was calculated using a bivariate logistic regression model in which receipt of combination therapy was the outcome variable.

All statistical analyses were performed with the Intercooled Stata program, version 11.

## RESULTS

**Figure 1.**
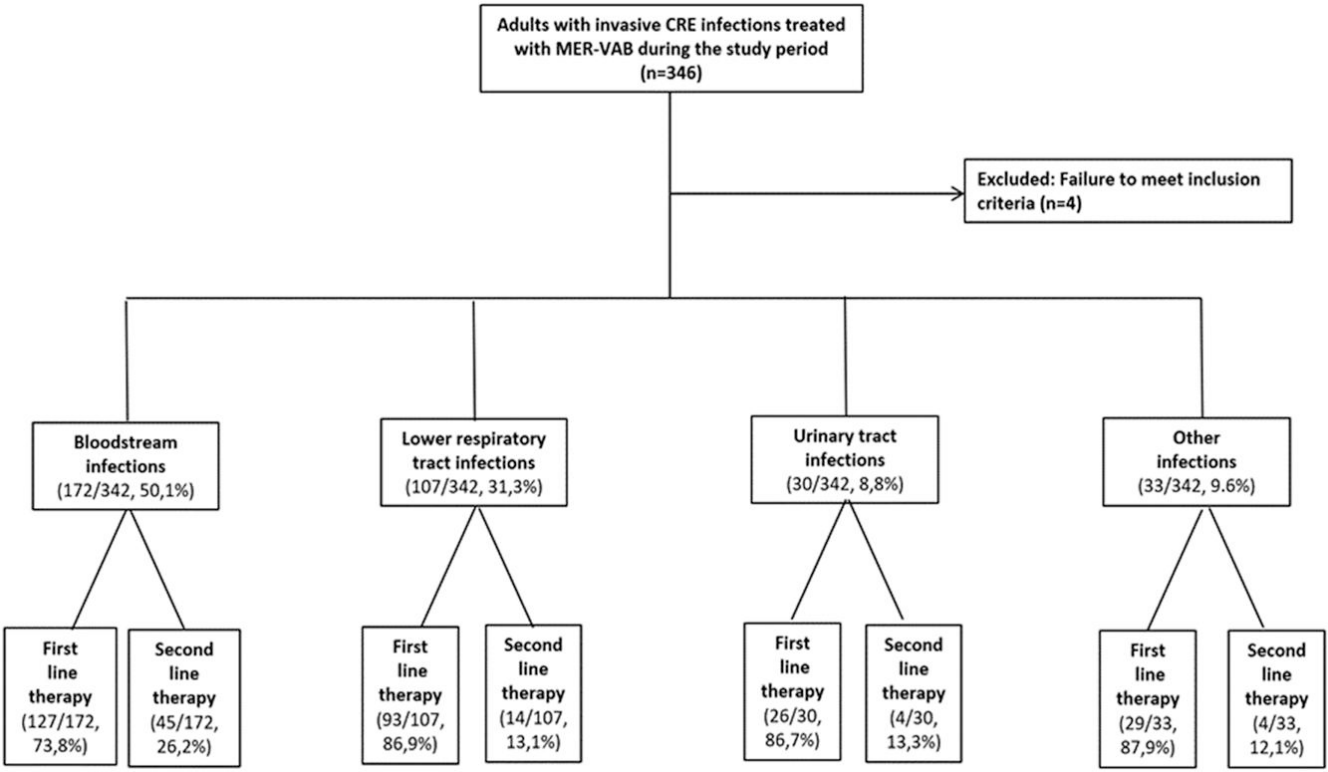
Flow chart showing cohort enrollment.

During the study period, 346 adults hospitalized in the participating centers received meropenem-vaborbactam. As summarized in Figure 1, the final cohort analyzed comprised 342 adults with KPC-Kp infections who received at least 24 hours of meropenem-vaborbactam therapy. All isolates were resistant to penicillins, extended-spectrum cephalosporins, ciprofloxacin, and meropenem, and 85 (24.8%) were resistant to ceftazidime-avibactam.

Table 1 outlines the characteristics of patients with KPC-Kp infections treated with meropenem-vaborbactam. Patients ranged in age from 27 to 89 years and two-thirds were male (69.6%). Most infections (318/342, 92.9%) were hospital-acquired. Almost half (161/342, 47.1%) were diagnosed during an intensive care unit (ICU) stay, and 125 (36.5%) were in a medical ward. COVID-19 was the underlying condition for admission in 21.1% of patients (72/342).

**Table 1.** Characteristics of Patients With Meropenem-Vaborbactam-Treated KPC-Kp Infections

Variable	All Infections (n = 342)	BSIs (n = 172)	LRTI (n = 107)	cUTI (n = 30)	Other^a^ (n = 33)
Patient variables					
Males	238 (69.6)	127 (73.8)	77 (71.9)	16 (53.3)	18 (54.5)
Age—median (IQR)	67 (58–75)	66 (56–75)	66.5 (58–74)	73.5 (68–79)	65 (54–75)
Comorbidities	…	…	…	…	…
COPD	50 (14.6)	16 (9.3)	25 (23.4)	6 (20.0)	3 (9.1)
Cardiovascular disease	185 (54.1)	99 (57.6)	51 (47.7)	19 (63.3)	16 (48.5)
Cerebrovascular disease or dementia	56 (16.4)	31 (18.1)	16 (14.9)	5 (16.7)	4 (12.1)
Solid tumor	70 (20.5)	39 (22.7)	18 (16.8)	5 (16.7)	8 (24.2)
Hematologic malignancy	18 (5.3)	10 (5.8)	6 (5.6)	1 (3.3)	1 (3.1)
Liver disease	36 (10.5)	19 (11.1)	14 (13.1)	2 (6.7)	1 (3.1)
Immunodeficiency	20 (5.9)	10 (5.8)	4 (3.7)	3 (10.0)	3 (9.1)
Solid organ transplantation	40 (11.7)	23 (13.4)	11 (10.3)	1 (3.3)	5 (15.2)
Chronic renal failure	72 (21.1)	34 (19.8)	11 (10.3)	14 (46.7)	13 (39.4)
Diabetes mellitus	77 (22.5)	29 (16.9)	32 (29.9)	6 (20.0)	10 (30.3)
Neutropenia	8 (2.3)	4 (2.3)	4 (3.7)	0	0
Charlson Comorbidity Index ≥ 3	272 (79.5)	139 (80.8)	84 (78.5)	25 (83.3)	24 (72.7)
Preinfection health care interventions	…	…	…	…	…
Previous hospital admission^b^	193 (56.4)	102 (59.3)	52 (48.6)	19 (63.3)	20 (60.6)
Surgery^c^	120 (35.1)	68 (39.5)	27 (25.2)	6 (20.0)	19 (57.6)
Dialysis^c^	39 (11.4)	22 (12.8)	11 (10.3)	2 (6.7)	4 (12.1)
Endoscopy^d^	45 (13.2)	28 (16.3)	9 (8.4)	2 (6.7)	6 (18.2)
Mechanical ventilation^d^	143 (41.8)	60 (34.8)	76 (71.1)	1 (3.3)	6 (18.2)
Indwelling devices	…	…	…	…	…
Central venous catheter^d^	212 (61.9)	105 (61.1)	82 (76.6)	5 (16.7)	20 (60.6)
Bladder catheter^d^	234 (68.4)	115 (66.9)	93 (86.9)	11 (36.7)	15 (45.5)
Nasogastric tube^d^	118 (34.5)	52 (30.2)	57 (53.3)	4 (13.3)	5 (15.2)
Surgical drain^d^	77 (22.5)	42 (24.4)	14 (13.1)	2 (6.7)	19 (57.6)
Infection characteristics	…	…	…	…	…
Hospital-acquired	318 (92.9)	159 (92.4)	103 (96.3)	26 (86.7)	30 (90.9)
LOS before infection	16 (9–32)	16 (9–35)	15 (8–27)	9.5 (9–46)	17 (10–34)
LOS after infection	18 (10–42)	18.5 (11–34.5)	18 (10–47)	20 (10–54)	15 (10–39)
COVID-19	72 (21.1)	20 (11.6)	46 (42.9)	4 (13.3)	2 (6.1)
Severity of illness^e^	…	…	…	…	…
INCREMENT score ≥8	156 (45.6)	81 (47.1)	54 (50.5)	5 (16.7)	16 (48.5)
Septic shock	51 (14.9)	30 (17.4)	10 (9.4)	5 (16.7)	6 (18.2)
Ward submitting index culture	…	…	…	…	…
Medical	125 (36.5)	70 (40.7)	23 (21.5)	21 (70.0)	11 (33.3)
Surgical	49 (14.3)	23 (13.4)	4 (3.7)	6 (20.0)	16 (48.5)
ICU	161 (47.1)	74 (43.1)	79 (73.8)	2 (6.7)	6 (18.2)
CAZ-AVI resistance	85 (24.8)	45 (26.2)	23 (21.5)	9 (30.0)	8 (24.2)

Unless otherwise stated, data are expressed as numbers (%).

Abbreviations: BSI, bloodstream infection; CAZ-AVI, ceftazidime-avibactam; COPD, chronic obstructive pulmonary disease; cUTI, complicated urinary tract infection; CU, intensive care unit; IQR, interquartile range; LOS, length of stay; LRTI, low respiratory tract infections.

^a^Other: 17 abdominal infections, 7 central nervous system infections, 5 osteoarticular infections, 4 skin and soft tissue infections.

^b^During the 6 mo preceding infection onset.

^c^During the 30 days preceding infection onset.

^d^At any time during the 120 h preceding infection onset.

^e^At infection onset.

A total of 172 of the infections (50.3%) were BSIs, and 170 (49.7%) were nonbacteremic infections: 107 (31.3%) were lower respiratory tract infections (LRTIs), 30 (8.8%) were complicated urinary tract infections (cUTIs), and 33 (9.6%) were infections involving other sites. Among LRTIs, 76/107 (71.1%) were ventilator-associated pneumonia, and among BSIs, 43/172 (25.0%) were catheter-related. Almost half of the KPC-Kp infections (n = 156, 45.6%) were classified as high mortality risk according to INCREMENT score.

### Treatment Regimens

As shown in Table 2, the median duration of meropenem-vaborbactam therapy was 11 days (interquartile range: 7-14 days). Meropenem-vaborbactam was started within 48 hours of infection onset in 148 patients (43.3%), most of which were bacteremic. One hundred and ninety-four (56.7%) patients received meropenem-vaborbactam after 48 hours, and 67 of them as a second-line therapy after an initial therapy with other active drugs (ceftazidime-avibactam, cefiderocol, or colistin).

**Table 2.** Meropenem-Vaborbactam Treatment Features and Outcomes

Variable	All Infections (n = 342)	BBSIs (n = 172)	LRTI (n = 107)	cUTI (n = 30)	Other^a^ (n = 33)
MER-VAB treatment variables	…	…	…	…	…
Days of treatment—median (IQR)	11 (7–14)	11 (7–15)	11 (7–14)	7 (3–11)	14 (9–21)
Started empirically	47 (13.7)	18 (10.5)	23 (21.5)	3 (10.0)	3 (9.1)
Started within 48 h of infection onset	148 (43.3)	64 (37.2)	51 (47.7)	19 (63.3)	14 (42.4)
Started as second-line therapy	67 (19.6)	45 (26.2)	14 (13.1)	4 (13.3)	4 (12.1)
Monotherapy regimens	213 (62.3)	109 (63.4)	74 (69.2)	12 (40.0)	18 (54.5)
Combination regimens with:	129 (37.7)	63 (36.6)	33 (30.8)	18 (60.0)	15 (45.5)
1 other active antimicrobial	98 (28.6)	47 (27.3)	24 (22.4)	18 17 (56.7)	9 (27.3)
≥2 active antimicrobials	32 (9.4)	16 (9.3)	9 (8.4)	1 (3.3)	6 (18.2)
Dose adjusted for renal function	101 (29.5)	50 (29.1)	27 (25.2)	13 (43.3)	11 (33.3)
Prolonged infusion	231 (67.5)	123 (71.5)	68 (63.5)	21 (70.0)	19 (57.6)
Outcomes^b^	…	…	…	…	…
30-d all-cause mortality	108 (31.6)	57 (33.2)	35 (32.7)	4 (13.3)	12 (36.4)
Infection relapse^c^	33 (9.6)	18 (10.5)	10 (9.4)	4 (13.3)	1 (3.1)
Development of in vitro MER-VAB resistance	6 (1.7)	2 (1.2)	3 (2.8)	…	1 (3.1)
Adverse reactions	2 (0.6)	0	1 (0.9)	1 (3.3)	0

Unless otherwise stated, data are expressed as numbers (%).

Abbreviations: BSI, bloodstream infection; cUTI, complicated urinary tract infection; IQR, interquartile range; LRTI, low respiratory tract infections; MER-VAB, meropenem-vaborbactam.

^a^Other: 17 abdominal infections, 7 central nervous system infections, 5 osteoarticular infections, 4 skin and soft tissue infections.

^b^Assessed during the index hospitalization.

^c^Diagnosed microbiologically during the index hospitalization after the original infection had been classified as microbiologically and/or clinically cured.

In almost one third of patients (101/342, 29.5%), dosage adjustments for impaired renal function were performed. More than 60% of all infections were managed with meropenem-vaborbactam monotherapy. Combination regimens, which generally consisted of meropenem-vaborbactam plus 1 other active drug (usually fosfomycin, tigecycline, or gentamicin) were used in 129 patients (37.7%). Prolonged infusion was used in more than half of patients (231/342, 67.5%).

### Outcomes

Thirty days after onset of infections, 108/342 (31.6%) of patients had died: 33.2% (57/172) of those with BSI, 32.7% (35/107) with LRTI, 13.3% (4/30) with cUTI, and 36.4% (12/33) with other infections. The mortality rate of bacteremic LRTI is 47.4% (27/57).

**Figure 2.**
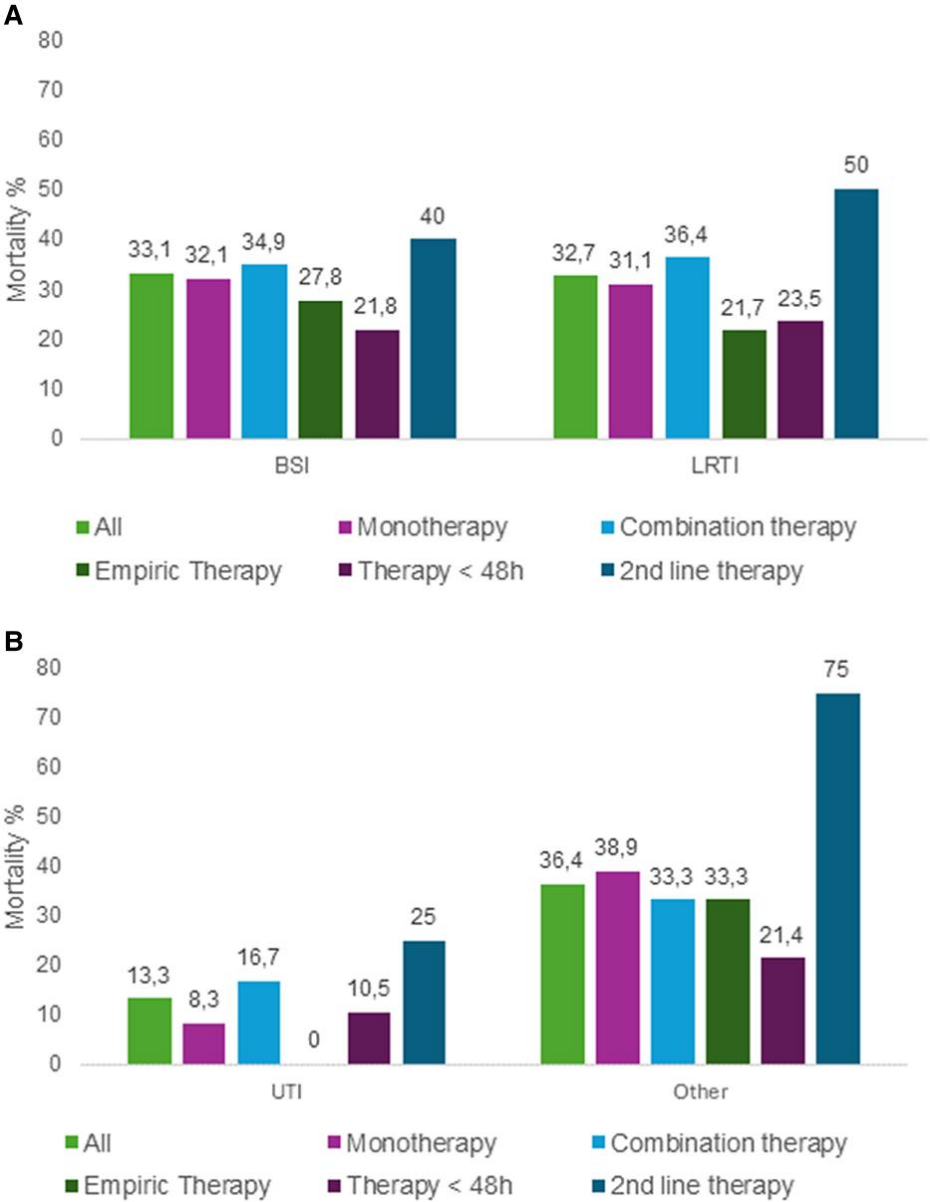
Thirty-day mortality rates in patients receiving meropenem-vaborbactam alone, or in combination with other active antimicrobials, started as empiric therapy, or within 48 h from index culture or as second-line therapy. Results are shown for (*A*) patients with BSIs (n = 170) and LRTI (n = 104); (*B*) patients with complicated urinary tract infections (cUTIs, n = 25), and infections at other sites (n = 33).

The 30-day mortality rate was 30.9% (66/213) for patients treated with monotherapy, and 32.6% (42/129) for those that received combination regimens. When meropenem-vaborbactam was started within 48 hours from the onset of infection, the mortality was 20.9% (31/148). Mortality rates for patients who received meropenem-vaborbactam as a second-line therapy was 43.3% (29/67). In a subgroup of patients with infections caused by ceftazidime-avibactam-resistant isolates, the mortality rate was 24.2% (8/33) for patients treated with meropenem-vaborbactam as first-line therapy and 36.5% (19/52) for those that received meropenem-vaborbactam as second-line therapy. Figure 2 shows mortality data of patients who received meropenem-vaborbactam alone, or in combination with other active antimicrobials, started as empiric therapy, or within 48 hours from index culture or as second-line therapy.

**Figure 3.**
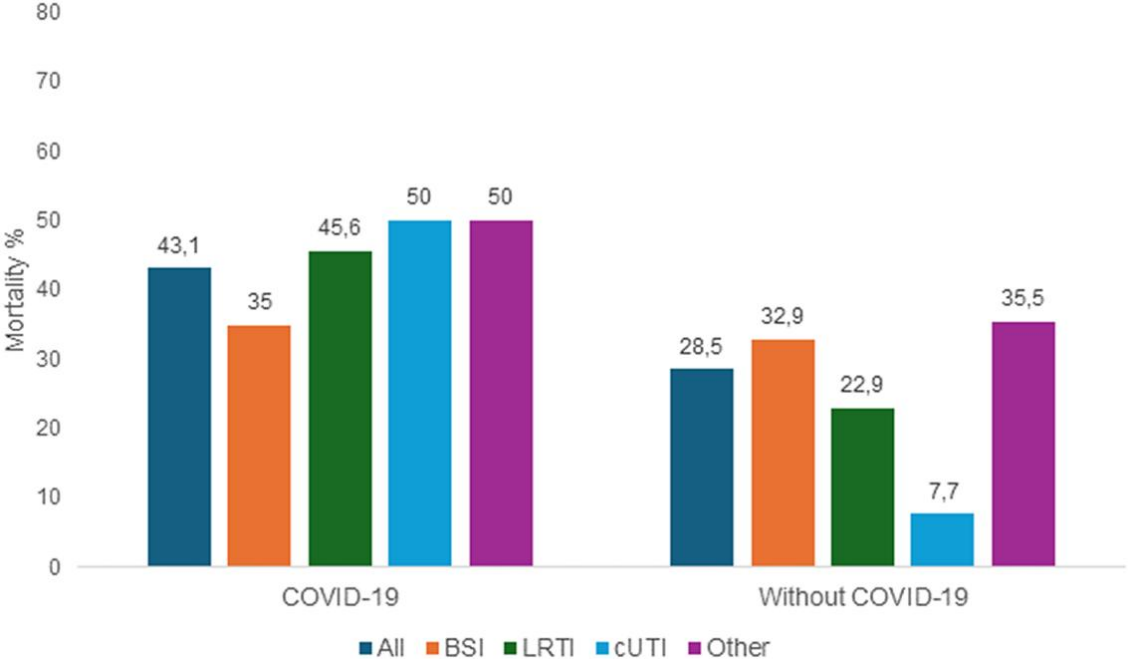
Thirty-day mortality rates in patients receiving meropenem-vaborbactam for different infections depending on whether there is an underlying COVID-19 or not. BSI, bloodstream infection; LRTI, lower respiratory tract infection; cUTI, complicated urinary tract infection; Other, infections involving other sites.

In the subgroup of patients with underlying COVID-19, the overall mortality was 43.1% (31/72), whereas in non-COVID-19 patients it was 28.5% (77/270). COVID-19 significantly decreased survival in patients with LRTI 45.6% (21/46), and cUTI 50% (2/4) (Figure 3).

Thirty-three of 342 (9.6%) patients (18 with BSIs, 10 with LRTIs, 4 with cUTIs, and 1 with another type of infection) experienced clinical relapses 16 to 22 days (median, 18 days) after meropenem-vaborbactam was discontinued. In 27 of these 33 cases, the KPC-Kp isolate recovered during the relapse displayed persistent in vitro susceptibility to meropenem-vaborbactam, and microbiological and/or clinical cures were achieved after retreatment with meropenem-vaborbactam as monotherapy in all but 4 patients treated with meropenem-vaborbactam plus fosfomycin. In the remaining 6 relapses, the KPC-Kp strain had become resistant to meropenem-vaborbactam. No statistically significant relationship was observed between relapse and the use of meropenem-vaborbactam monotherapy versus combination regimens.

Adverse reactions were observed in 0.6% (2/342) of the patients (rash in 1, nausea and vomiting in 1). All outcomes observed during the index hospitalization are shown in Table 2.

### Mortality Predictors

Univariate analysis revealed significant differences between the survivor and nonsurvivor subgroups (Table 3). A significantly higher percentage of patients of the latter group were admitted to the ICU, on dialysis treatment, had hospital-acquired infections, indwelling nasogastric tube, and received meropenem-vaborbactam as a second-line therapy. Nonsurvivors also had significantly higher mean Charlson Comorbidity Index and INCREMENT score at infection onset and infection presentation that included septic shock.

**Table 3.** Univariate Analysis of Factors Associated With 30-d Mortality

	No. (%) of Patients		*P* Value	OR (95% CI)
Variable	Nonsurvivors	Survivors		
	n = 108 (31.6)	n = 234 (68.4)		
Patient variables				
Male	70 (64.8)	168 (71.8)	.19	0.72 (0.43–1.22)
Age—median (IQR)	69 (60.5–75)	67 (56–74)	.13	-
Comorbidities	…	…	…	…
COPD	13 (12.1)	37 (15.8)	.36	0.73 (0.34–1.48)
Cardiovascular disease	52 (48.2)	133 (56.8)	.14	0.70 (0.43–1.15)
Cerebrovascular disease or dementia	18 (16.7)	38 (16.2)	.92	1.03 (0.52–1.97)
Solid tumor	19 (15.6)	51 (21.8)	.37	0.77 (0.40–1.41)
Hematologic malignancy	7 (6.5)	11 (4.7)	.49	1.40 (0.45–4.10)
Liver disease	14 (12.9)	22 (9.4)	.31	1.44 (0.65–3.08)
Immunodeficiency	6 (5.6)	14 (5.9)	.87	0.92 (0.28–2.66)
Solid organ transplantation	13 (12.1)	27 (11.5)	.89	1.05 (0.47–2.21)
Chronic renal failure	20 (18.5)	52 (22.2)	.43	0.79 (0.42–1.45)
Diabetes	25 (23.2)	52 (22.2)	.85	1.05 (0.58–1.87)
Neutropenia	2 (1.8)	6 (2.6)	.68	0.72 (0.07–4.09)
Charlson Comorbidity Index ≥ 3	96 (88.9)	176 (75.2)	.003	2.64 (1.32–5.65)
Ward submitting index culture	…	…	…	…
Medical	37 (34.3)	88 (37.6)	.55	0.86 (0.52–1.43)
Surgical	9 (8.3)	40 (17.1)	.03	0.44 (0.18–0.97)
ICU	62 (57.4)	99 (42.3)	.009	1.84 (1.13–2.99)
Preinfection health care interventions				
Surgery^a^	34 (31.5)	86 (36.7)	.34	0.79 (0.47–1.32)
Dialysis^a^	20 (18.5)	19 (8.1)	.004	2.57 (1.23–5.35)
Endoscopy^b^	12 (11.1)	33 (14.1)	.44	0.76 (0.34–1.59)
Mechanical ventilation^b^	55 (50.9)	88 (37.6)	.02	1.72 (1.06–2.80)
Indwelling devices	…	…	…	…
Central venous catheter^b^	72 (66.7)	140 (59.8)	.23	1.34 (0.81–2.24)
Bladder catheter^b^	74 (68.5)	160 (68.8)	.97	1.00 (0.60–1.70)
Nasogastric tube^b^	50 (46.3)	68 (29.1)	.001	2.10 (1.27–3.46)
Surgical drain^b^	27 (25.0)	50 (21.4)	.45	1.23 (0.69–2.16)
Infection characteristics				
Hospital-acquired	106 (98.1)	212 (90.6)	.01	5.50 (1.31–48.96)
BSIs	57 (52.8)	115 (49.2)	.53	1.16 (0.71–1.87)
LRTIs	35 (32.4)	72 (30.7)	.76	1.08 (0.64–1.81)
cUTIs	4 (3.7)	26 (11.1)	.02	0.31 (0.07–0.92)
Other	12 (11.1)	21 (8.9)	.53	1.27 (0.54- 2.83)
Disease severity of illness^c^	…	…	…	…
INCREMENT score ≥8	75 (69.4)	81 (34.6)	<.001	4.29 (2.56–7.24)
Septic shock	35 (32.4)	16 (6.8)	<.001	6.53 (3.28–13.39)
COVID-19	31 (28.7)	41 (17.5)	.01	1.89 (1.06–3.34)
MER-VAB treatment variables	…	…	…	…
Started empirically	11 (10.2)	36 (15.4)	.19	0.62 (0.27–1.32)
Started within 48 h of infection onset	31 (28.7)	117 (50.0)	<.001	0.40 (0.24–0.67)
Started as second-line therapy	29 (26.8)	38 (16.2)	.02	1.89 (1.05–3.39)
Monotherapy regimens	66 (61.1)	147 (62.8)	.76	0.93 (0.57–1.53)
Combination regimens with:	42 (38.9)	87 (37.2)	.76	1.07 (0.65–1.76)
1 other active drug	28 (25.9)	69 (29.5)	.49	0.84 (0.48–1.44)
≥ 2 other active drug	14 (12.9)	18 (7.7)	.12	1.79 (0.79–3.97)
Dose adjusted for renal function	38 (35.2)	63 (26.9)	.12	1.47 (0.87–2.47)
Prolonged infusion	70 (64.8)	161 (68.8)	.46	0.83 (0.50–1.39)
Outcomes^d^	…	…	…	…
Infection relapse^e^	11 (10.2)	22 (9.4)	.82	1.09 (0.46–2.46)
Development of in vitro MER-VAB resistance	4 (3.7)	2 (0.8)	.06	4.46 (0.63–49.80)
Adverse reactions	1 (0.9)	1 (0.4)	.57	2.18 (0.03–171.64)

Data are expressed as numbers (%) unless otherwise stated.

Abbreviations: BSI, bloodstream infection; COPD, chronic obstructive pulmonary disease; cUTI, complicated urinary tract infection; ICU, intensive care unit; IQR, interquartile range; LRTI, low respiratory tract infections; MER-VAB, meropenem-vaborbactam; OR, odds ratio.

^a^During the 30 days preceding infection onset.

^b^During the 72 h preceding infection onset.

^c^At infection onset.

^d^Assessed during the index hospitalization.

^e^Diagnosed microbiologically during the index hospitalization after microbiological and/or clinical cure of the original infection.

Patients who survived were more frequently admitted to surgical wards, affected by cUTI, and treated with meropenem-vaborbactam within 48 hours from infection onset.

In a multiple Cox regression model (Table 4), 30-day mortality was independently associated with septic shock at infection onset, Charlson Comorbidity Index ≥ 3, dialysis, concomitant COVID-19, and INCREMENT score ≥ 8. Administration of meropenem-vaborbactam within 48 hours from infection onset was a negative predictor of mortality. All predictors except administration of meropenem-vaborbactam within 48 hours remained significant when the multiple Cox regression model was repeated after adjustment for the propensity score for receipt of combination therapy.

**Table 4.** Multivariate Analysis of Factors Associated With 30-d Mortality

	Adjusted For The Propensity Score Matching For Combination Therapy			
	No		Yes	
Variables	*P* value	HR (95% CI)	*P* value	HR (95% CI)
Septic shock at infection onset	<.001	3.65 (2.27–5.87)	<.001	2.85 (1.65–4.92)
Charlson Comorbidity Index ≥ 3	.005	2.42 (1.31–4.47)	.01	2.33 (1.22–4.48)
Dialysis^a^	.04	1.69 (1.03–2.78)	.02	1.91 (1.11–3.31)
COVID-19	.03	1.64 (1.05–2.56)	.04	1.62 (1.19–2.63)
INCREMENT score ≥8	.04	1.65 (1.02–2.67)	.01	2.02 (1.13–3.61)
MER-VAB started within 48 h of infection onset	.05	0.69 (0.55–1.06)	.39	0.81 (0.52–1.29)

Abbreviations: CI, confidence interval; MER-VAB, meropenem-vaborbactam; HR, hazard ratio.

## DISCUSSION

To date, this is the most extensive study evaluating real-world, postmarketing efficacy of meropenem-vaborbactam therapy for KPC-Kp infections. The overall 30-day mortality rate is 31.6% despite almost half of the patients being at high mortality risk with INCREMENT score ≥8 or admitted to the ICU at the onset of infection.

The mortality rate in our cohort is higher compared to previously published studies. In the TANGO II clinical trial, the 28-day mortality was 15.6% for meropenem-vaborbactam compared with a 33.3% for the comparator group [8]. In other postmarketing real-world observational studies, the mortality rate ranged between 7.5% and 24.3% [9–14]. In addition, in a very recent published paper, the overall 30-day mortality rate was 15.4%, although only 60% of patients had a CRE infection [15].

No significant survival benefit was observed when meropenem-vaborbactam was administered in combination with another active agent supporting the use of meropenem-vaborbactam monotherapy, as suggested by the results of the TANGO II study [8]. Given the risks associated with the unnecessary use of antibiotics, it is important to highlight that meropenem-vaborbactam proves effective when employed as monotherapy.

Interestingly, our study confirms that the outcome of patients treated with meropenem-vaborbactam as first-line therapy for infections caused by isolates resistant to ceftazidime-avibactam is comparable to that of patients with infections susceptible to ceftazidime-avibactam, as already reported in a smaller number of cases [12]. It is worth noting that the high proportion of patients with ceftazidime-avibactam-resistant infections in our study is probably mainly the result of selection bias, as in clinical practice meropenem-vaborbactam is often used to treat infections caused by resistant ceftazidime-avibactam strain. Although comprehensive nationwide data about the prevalence of KPC-Kp ceftazidime-avibactam-resistant infections are not available, reports from other Italian studies suggest that less than 10% of KPC-Kp are resistant to ceftazidime-avibactam [5, 22].

Notably, the mortality rate in our study is higher among patients with underlying COVID-19, with relevant numbers in LRTI with a mortality rate of 43%. This aligns with previous reports indicating an augmented mortality risk because of the coexistence of hospital-acquired infections in COVID-19 patients admitted to the ICU [23–25]. Conversely, in patients without COVID-19, the mortality rate for LRTIs treated with meropenem-vaborbactam is 23%. While acknowledging the challenge of comparing cohorts, one might speculate that this figure is lower than what has been previously reported in other real-world studies evaluating patients with LRTIs treated in the pre-COVID-19 era with ceftazidime-avibactam, the most commonly used drug for these infections [12, 22, 26, 27]. The seemingly enhanced efficacy of meropenem-vaborbactam in LRTIs may find support in studies investigating the pharmacokinetic/pharmacodynamic target attainment and microbiological outcomes of meropenem-vaborbactam in treating documented KPC-Kp pneumonia, including cases of ventilator-associated pneumonia [28, 29].

However, the lower mortality rate in patients treated with meropenem-vaborbactam does not seem to extend to infections other than LRTIs and should be further confirmed by prospective studies designed for this specific comparison [12, 22, 26, 27].

Numerous studies have emphasized the time-sensitive nature of treating serious infections, with delays in appropriate therapy carrying negative consequences [30–32]. In fact, using meropenem-vaborbactam as second-line therapy after the failure of initial treatment with another active drug was linked to a mortality rate exceeding 40%, suggesting that factors associated with treatment failure in severe clinical conditions may persist even when changing antibiotics.

Consistent with other real-world studies on patients with KPC-Kp infections treated with meropenem-vaborbactam, recurrence rates in our study were low (9.6%), with only 5 relapses where the KPC-Kp strain had developed resistance to meropenem-vaborbactam [10–12, 14].

Our results highlight the potential adverse effects of dialysis for impaired renal function on outcomes, as indicated by Crass et al. Their observations emphasize the limitations of protocols for adjusting antibiotic dosages based on renal function, especially for antibiotics with wide therapeutic indices, as they predominantly rely on data from individuals with stable chronic kidney disease. Consequently, such dosages may not be suitable for antibiotic treatments during severe infectious events, often associated with transient acute kidney injury [33].

The main limitation of our study is its retrospective observational design, with intrinsic limitations in terms of patient selection and timing of antibiotic initiation. Moreover, the retrospective cohort included several different subgroups of patients, making the population heterogeneous and limiting; therefore, the comparison with other studies or the translation of our study for specific categories of patients. In addition, the presence of a considerable number of patients with concomitant COVID may have influenced mortality rate.

In conclusion, the data derived from this extensive multicenter cohort lend additional support to the efficacy of meropenem-vaborbactam in treating severe KPC-Kp infections, even when used as monotherapy.

## Notes

***Financial support*.** The research was supported by EU funding within the Next Generation EU-MUR PNRR “Extended Partnership initiative on Emerging Infectious Diseases” (Project no. PE00000007, INF-ACT) and “Tuscany Health Ecosystem” (Project no ECS00000017—THE)

***Patient consent statement*.** The protocol received approval from the Research Ethics Committee of the Coordinating Center (Regione Toscana, Area vasta sud est) Prot n 21874, and the informed consent requirement was waived because of the retrospective and noninterventional nature of the study according to AIFA Guidelines (AIFA 20/03/08—C.U.R.I. n 76 del 31/03/2008) and EU Regolation 2016/679 (General Data Protection Regulation).
